# Functional 3D-Printed Polymeric Materials with Metallic Reinforcement for Use in Cut-Resistant Gloves

**DOI:** 10.3390/ma17010090

**Published:** 2023-12-23

**Authors:** Emilia Żyłka, Emilia Irzmańska, Jakub Saramak, Magdalena Jurczyk-Kowalska

**Affiliations:** 1 Central Institute for Labour Protection—National Research Institute, Department of Personal Protective Equipment, Wierzbowa 48, 90-133 Lodz, Poland; emirz@ciop.lodz.pl; 2 SMK3D Company, Opałowa 11/17, 93-487 Lodz, Poland; saramakjakub@gmail.com; 3 Faculty of Material Science and Engineering, Warsaw University of Technology, Wołoska 141, 02-507 Warsaw, Poland; magdalena.jurczyk@pw.edu.pl

**Keywords:** polymeric materials, polymer composites, 3D printing technology, metallic reinforcement, protective gloves, cut resistance materials

## Abstract

Given the mechanical hazards occurring in the workplace, cut resistance is a particularly important protective parameter. 3D printing is an innovative technology that has recently garnered great interest. It enables the creation of functional polymeric materials with metal reinforcement for use in cut-resistant gloves. The present study characterized and tested 3D-printed polymeric materials intended for such applications. The materials were made from commercially available 3D printing polymer filaments. Metallic reinforcement (stainless steel wire with a diameter of 0.04 mm) was added to the two selected materials (thermoplastic polyurethane and FiberFlex30D). Tests have shown that materials containing metallic reinforcement demonstrate higher mechanical resistance. Cut resistance increased by 70%, and the force needed to tear the sample increased by over 20% compared to the pure polymer. The presence of metallic reinforcement strengthens the structure of the material and changes the cracking mechanism. The tearing occurs in the test area, not in the bell area. These findings demonstrate the feasibility of applying functional 3D-printed polymeric materials with metal reinforcement in cut-resistant gloves.

## 1. Introduction

Personal protective equipment (PPE) consists of devices that protect workers against the hazards that they face while performing their jobs. Injuries to the upper limbs are the most common type of accidents at work that occur while performing manual tasks [1]. In order for protective gloves to effectively prevent mechanical injuries, they need to meet a number of protective requirements imposed by Regulation (EU) 2016/425 of the European Parliament and of the Council of 9 March 2016 on personal protective equipment and repealing Council Directive 89/686/EEC (2016). Regarding mechanical hazards, a particularly important protective parameter is cut resistance. The basic standard specifying the requirements for gloves protecting against mechanical factors, including cuts, is EN 388:2016+A1:2018 [2], while materials with high cut resistance should be tested in accordance with EN ISO 13997:1999 [3]. Performing both tests produces more consistent results by reducing the impact of blade blunting [4].

Recently, there has been high market demand for hand protection products intended for workers exposed to direct contact with sharp and rough surfaces in a variety of industries. Due to technological advancement, 3D printing is widely used to produce mechanically resistant structures of all kinds of geometry [5,6]. Thus, there is growing interest in the feasibility of using this technology to produce highly mechanically resistant structures. Moreover, 3D printing is innovative and has become a versatile technological tool [7,8]. Rapid prototyping, the ability to print large structures, a reduction in print defects, and improved mechanical properties are some of the key factors that are driving the development of additive manufacturing (AM) technology [9]. The range of materials used in 3D printing includes thermoplastics, ceramics, graphene-based materials, and metals. The period related to the COVID-19 pandemic resulted in an increase in demand for personal protective equipment [10,11]. Obtaining materials using 3D printing provided quick and easy access to personal protective equipment. This resulted in an increase in interest in using this technology in this area. New polymeric, metal, ceramic, and composite materials are being developed, enabling new applications. One of the interesting directions of 3D printing may be the production of hybrid composite materials used in personal protective equipment. There are many literature reports on hybrid materials reinforced with metal oxides, carbon nanotubes, and metallic elements. They have a great potential to replace, e.g., metals in many applications such as transport, shipping, and construction [12]. It should be emphasized that to create a hybrid material, individual components obtained from a 3D printer must be combined. For this purpose, manual layering [13], mechanical methods [14], or the lamination of several layers at the same time have been used [15,16]. Unfortunately, all of these processes are expensive and time-consuming.

An interesting option is the use of 3D printing to incorporate metallic reinforcement into the polymer matrix to enhance the mechanical and strength properties. Doner et al. [17] introduced metallic iron reinforcement into a mixture of acrylonitrile-butadiene-styrene (ABS) polymers and thermoplastic polyurethane. The researchers found that the glass transition and melting temperatures were slightly higher for the particulate-reinforced samples as compared to the unreinforced ABS/TPU blend control. According to the authors, this was due to the fact that inclusions limited the mobility of the structure, and so more energy was needed to break down the molecular structure. Moreover, it was found that tensile strength and fracture toughness increased with the amount of iron powder up to 5%. Nikzad et al. [18] presented studies on the thermal and mechanical properties of ABS composites filled with iron or copper particles with a metal content of up to 40%. Using dynamic mechanical analysis, the authors demonstrated that the introduction of metallic fillers can potentially promote the processing of these materials during 3D printing. It should be emphasized that due to the high content of metallic particles in the matrix of the composite material, the composites show higher stiffness than those made of pure polymers. According to the authors, this allows the composite to withstand higher pressures during injection molding. Ryder et al. [19] obtained a polymer-metal composite by dissolving ABS in acetone and adding 10–23% non-spherical stainless steel powder. The resulting material was extruded to obtain composite fibers that were used to print test samples. The results clearly showed that polymer-metal composites with appropriate percentages of stainless steel (up to 15%) can be produced without compromising mechanical properties. In addition, some additional properties of the structural element, such as magnetic effects, were obtained. Palmero et al. [20] used scanning electron microscopy (SEM) to perform the microstructural and morphological evaluation of ABS polymer composites reinforced with aluminum and stainless steel particles. Particle size and distribution were found to influence the physical properties of composites and fibers. The smaller the particle size and higher the size distribution measured for aluminum powder (as compared to stainless steel particles), the easier the production of continuous filament with an increased filling factor. According to the authors, the results provide an appropriate basis for the development and implementation of innovative materials in sectors such as aeronautics and cosmonautics. Hamidi et al. [21] used 3D printing to introduce copper and steel wire into thermoplastic materials: polylactide (PLA) and polyethylene terephthalate-modified glycol with the addition of carbon nanotubes (PETG CNT). They compared their mechanical properties with available theoretical models. According to the authors, the effectiveness of continuous fiber reinforcement depends on the matrix material and the adhesion between the fiber and the matrix due to the formation of voids. It should be emphasized that the reinforced polymers showed an increase in strength of over 70% and were characterized by high flexibility. Materials of this type could be used in the production of soft pneumatic actuators.

The literature contains promising examples of research results confirming the possibility of using 3D printing to produce hybrid composite materials. However, there is not much information related to their practical application, and the proposed methods have not been transferred to industry. Therefore, a series of polymeric materials were produced using 3D printing technology to increase the cut resistance of protective gloves. We selected the two most promising materials and incorporated metallic reinforcement in the form of stainless steel wire. The influence of metallic reinforcement was discussed, and the mechanical and strength parameters, including tensile resistance, were examined. By characterizing and understanding the properties of the 3D-printed materials, it will be possible to better determine and adapt specific properties in PPE applications, especially in cut-resistant protective gloves.

## 2. Materials and Methods

### 2.1. Materials

**Figure 1:**
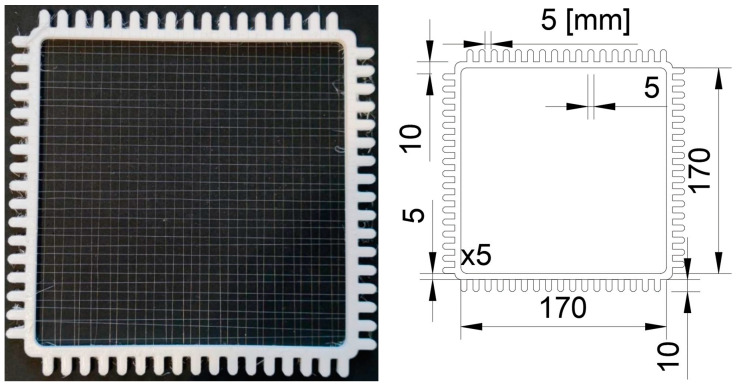
Metallic reinforcement inserted into polymer filaments during 3D printing. The marked distances in the drawing on the right are expressed in mm units.

The research materials consisted of two polymeric materials: thermoplastic polyurethane (TPU95A, Ultimaker, The Netherlands) and FiberFlex30D (FiberLab, Poland), which were prepared using 3D printing technology by the company SMK3D Jakub Saramak (Poland, Chechło Pierwsze). Test samples were printed using fused deposition modeling (FDM). Stainless steel 304L wire (Bekaert Fibre Technologies, Aalst, Belgium) with a diameter of 0.04 mm was incorporated into selected polymer filaments in the form of a mesh, as shown in Figure 1.

Special frames were designed to easily and precisely create 5 × 5 mm wire meshes. The frames had an internal area measuring 170 × 170 mm that enabled printing 100 × 100 mm samples. The frames were 5 mm thick, which ensured sufficient stiffness and prevented the wire from straining and deforming. Additionally, opposing teeth were staggered relative to each other by 5 mm to facilitate the process of mesh formation (Figure 1).

The materials (TPU95A and FiberFlex30D) were selected based on preliminary tests of the protective parameters of seven polymers with different physicochemical properties (Table S1). Test results for those materials in terms of cut, abrasion, puncture, and tear resistance are presented in the Supporting Information (Figures S1–S10). Given the thickness of the coatings most commonly used in commercially available protective gloves, two versions of samples—0.5 mm and 1.5 mm thick—were prepared. Most polymeric materials with a thickness of 0.5 mm achieved satisfactory protective parameters. However, from the standpoint of functional and economic aspects, it seems that such thin coatings might be quickly destroyed in the work environment. Among samples with a thickness of 1.5 mm, only those made of TPU95A and FiberFlex30D could be tested. The remaining materials showed high stiffness, making it impossible to mount them in the specimen holder. When considering the use of polymeric materials as anti-cut coatings, the aspect of ergonomics should also be taken into account. The glove must fit the hand and enable the performance of tasks in the work environment, which may be prevented by excessive material stiffness. In light of this, TPU95A and FiberFlex30D were selected for further tests.

### 2.2. Sample Preparation

Test samples were printed using fused deposition modeling (FDM). It is an additive manufacturing technique in which material is extruded through a nozzle and added to the growing workpiece to create a 3D object [22]. The devices used in this study are Ultimaker S5 (Ultimaker, The Netherlands) printers with 0.4 mm nozzles and a glass build plate, which ensures a smooth first layer [23]. Table 1 shows the printing and fan speeds as well as nozzle and build plate temperatures for specific materials.

The printing process was as follows: the first layer was 0.2 mm high, with the next nine being 0.15 mm high. After finishing the first layer, printing was paused for 20–60 s, and a framed wire mesh was applied to it, after which the printing was resumed. Such a short break time practically did not cause any reduction in the temperature of the nozzle and buildplate. Wires were embedded between the bottom 0.2 mm layer and the top two in the case of the 0.5 mm sample and nine 0.15 mm top layers in the case of the 1.5 mm sample. As a reminder, a wire with a diameter of 0.04 was four times smaller than the height of the layer, and thus, it deformed only one or two following layers.

### 2.3. Testing Methods

#### 2.3.1. Static Cut Resistance

**Figure 2:**
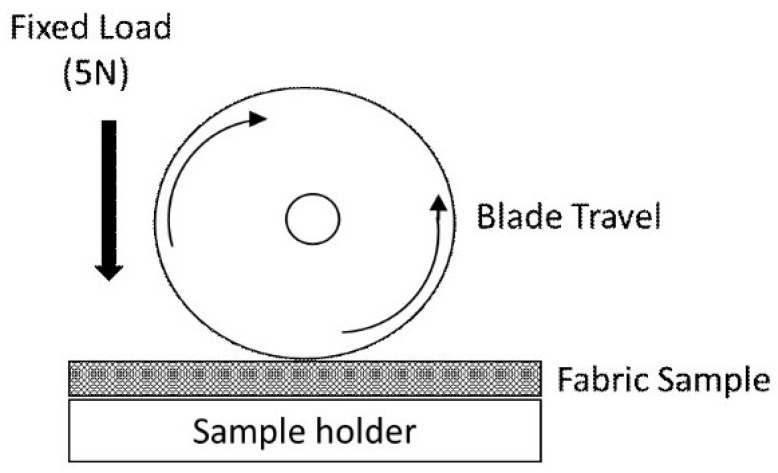
Type of blade used to test samples according to EN 388:2016+A1:2018.

Static cut resistance was evaluated according to the standard EN 388:2016+A1:2018 with a constant load applied to the blade, resulting in a force of (5 ± 0.5) N. During the test, a rotating circular blade was used with a diameter of (45 ± 0.5) mm, a thickness of (0.3 ± 0.03) mm, and a total cutting angle of 30–35° (Figure 2).

The blade shall be in stainless steel with a Vickers Hardness of 700 to 720. The horizontal movement was 50 mm long, with the blade rotating completely (360°) in the opposite direction. The sinusoidal cutting speed was (8 ± 2) cm/s. Test results were recorded in Table 2.

$\bar{C_{n}}$ represents the average number of cycles for the control specimen before and after the cut of the test specimen T_n_ and is calculated from the following Equation:

(1) $$\bar{C_{n}}=\frac{\left(C_{n}+C_{n+1}\right)}{2}$$

A unitless cut resistance index (I) was calculated for every test specimen according to Equation (2).

(2) $$I=\frac{1}{5}\sum_{n=1}^{5}i_{n}\;where\;i_{n}=\frac{\left(\bar{C_{n}}+T_{n}\right)}{\bar{C_{n}}}$$

The resulting index values were used to assign cut-resistant performance levels to the specimens, according to Table 3.

#### 2.3.2. Dynamic Cut Resistance

**Figure 3:**
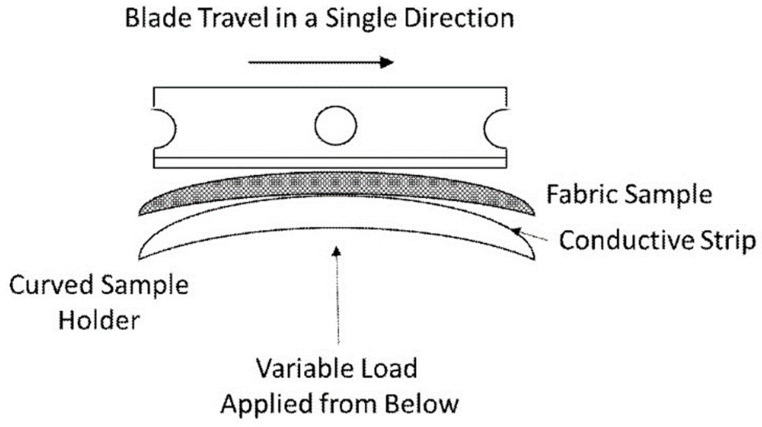
Type of blade used to test samples according to EN ISO 13997:1999.

Dynamic cut resistance was tested under variable load according to EN ISO 13997:1999 using an apparatus from P.I. Kontech (Łódź, Poland). Samples were mounted on a metal cylinder with a radius of (38 ± 0.5) mm, and a straight blade was drawn across the curvature of the cylinder with the plane of the blade at an angle of (90 ± 2)° to the long axis of the cylinder. The cutting edge of the blade was (74 ± 0.2) mm long, (18.5 ± 0.2) mm wide, and (1.0 ± 0.5) mm thick. The angle of the cutting edge was 22°. During the test, a variable force ranging from 1.0 N to 200.0 N was applied to the blade. The cutting rate was (2.5 ± 0.5) cm/s (Figure 3). Each result was corrected for blade sharpness. Performance levels were assigned to cutting force levels according to Table 4.

#### 2.3.3. Tensile Rest

Tensile testing was performed in accordance with EN 455-2:2015 [24] on selected samples to provide information on the mechanical properties and behavior of the material under tension. Samples were cut out in the shape of a dumbbell, 7.5 cm long and 4 mm wide, and clamped in the tester jaws under a pressure of 22 ± 5 kPa. The jaws advanced at a rate of 500 mm/min. The test was performed using an Instron universal tester.

### 2.4. Structural and Surface Examination Methods

Stereo microscopy

Sample morphology was characterized using an Opta-Tech SKMI20 stereo microscope at magnifications in the range of 7–45× and a ZEISS SteREO Discovery V8 (Opta-Tech, Warsaw, Poland) stereo microscope at the following magnifications: 0.3× (for a 2 mm marker), 0.5× (for a 1 mm marker) and 1× (for a 0.5 mm marker). In all cases, the samples were observed without any pre-treatment.

Scanning electron microscopy

The surface morphology of the samples was characterized using a HITACHI SU8000 (Hitachi, Tokyo, Japan) scanning electron microscope (SEM) operating at 5 kV. Specimens were cut from specific areas of the sample, attached to the microscope stage with conductive tape, and sputtered with gold. Observations were made using magnifications in the range of 50–2000×.

## 3. Results

Table 5 presents general views of the polymeric materials with metallic reinforcement as well as their macroscopic images at 15× magnification. The macroscopic images of the TPU95A sample show a clearly heterogeneous structure, with voids between individual print lines that may weaken the mechanical properties of the resulting material [25]. The implementation of metallic reinforcement was different for FiberFlex30D, in which case macroscopic images show a clearly homogeneous structure of the sample without longitudinal gaps between print lines.

**Figure 4:**
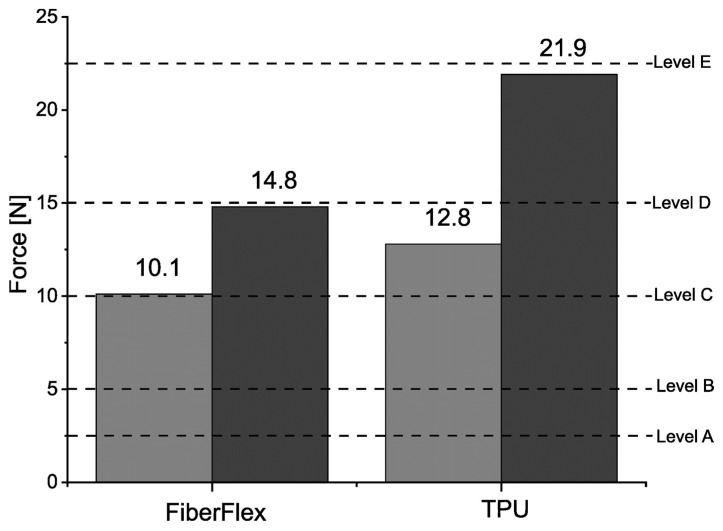
Cut resistance results for the tested materials according to EN ISO 13997:1999.

The cut resistance of the samples was tested with the TDM method, with the results shown in Figure 4 and summarized in Table 6. The reference samples were polymeric materials without metallic reinforcement. In the case of FiberFlex30D, the obtained performance levels of samples with and without reinforcement were the same, even though the strength of the reinforced ones increased by 60%. Of particular note is thermoplastic polyurethane. This unreinforced sample already obtained the highest performance level among all tested samples (C), and the implementation of metallic reinforcement increased its cut resistance by over 70%. The force needed to cut through it rose from 12.8 N to 21.9 N, and so the sample advanced to performance level D. It is clearly visible that the incorporation of metallic reinforcement considerably improved the cut resistance of the tested polymeric materials.

**Figure 5:**
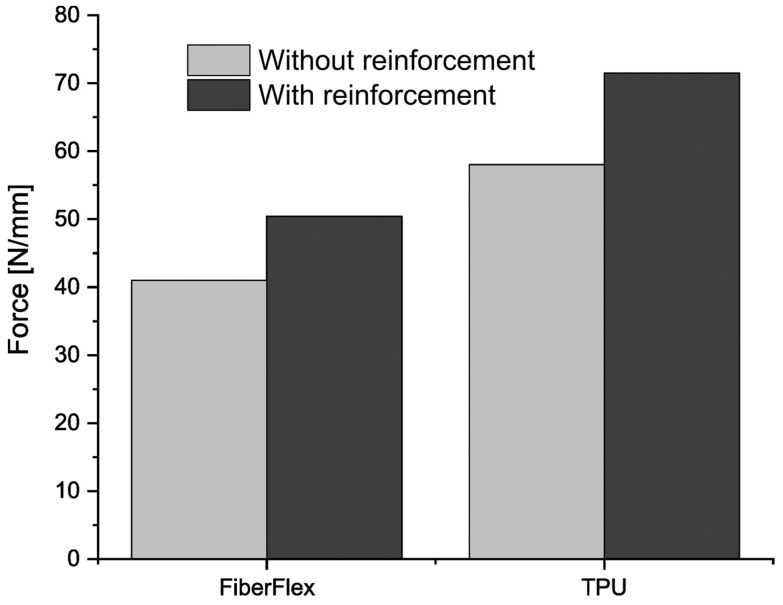
Results of the tearing force test according to EN 455-2:2015.

In order to determine the influence of metallic reinforcement on the mechanical parameters of the samples, tensile tests carried out according to EN 455-2:2015. Figure 5 shows a comparison of test results for unreinforced and reinforced samples. It is clearly visible that in both cases, the presence of metallic reinforcement significantly improved mechanical properties. The difference in breaking force increased by 20% and 25% for FiberFlex30D and TPU95A, respectively.

**Figure 6:**
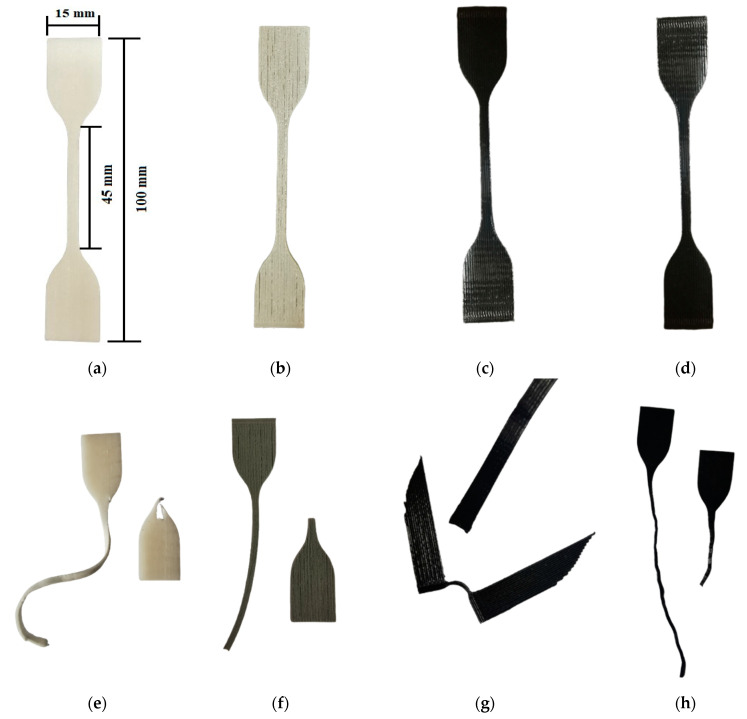
Images of samples pre-test: (**a**) TPU95A without reinforcement, (**b**) TPU95A with reinforcement, (**c**) FiberFlex30D without reinforcement, (**d**) FiberFlex with reinforcement; and post-test: (**e**) TPU95A without reinforcement, (**f**) TPU95A with reinforcement, (**g**) FiberFlex30D without reinforcement, (**h**) FiberFlex30D with reinforcement.

The samples before the test are shown in Figure 6. In order to determine the effect of reinforcement on the properties of the 3D-printed materials, images were acquired using a stereoscopic microscope and a scanning electron microscope (SEM). The former clearly shows differences in sample surface (Figures S11–S14). This is most likely due to the 3D printing technology itself and the types of materials used. The TPU95A sample changed its color as a result of stretching, which indicates a change in the direction of macromolecules as a result of elongation. In addition, the rupture of the sample did not occur in the neck area of the dumbbell sample but at the junction of the neck and bell areas of the sample (Figure 6e). The weakening of the material was most likely the result of discontinuities in the rupture area [26,27]. The presence of metallic reinforcement in the tested sample resulted in decreased elongation during the test. It should also be noted that another sample was damaged in the neck area (Figure 6f). The presence of the reinforcement changed the behavior of the sample during the test, strengthening the structure of the material. A similar effect was observed for the FiberFlex30D sample, where the tear occurred in the bell area (Figure 6g). In addition, the tested sample was not damaged by fracturing, but the tear occurred in the area of filament connection. Based on these observations, it can be concluded that the filaments were not well bonded to each other. Most probably, the parameters of the printing process did not ensure sufficient filament plasticization and did not fuse filaments [21,27]. The presence of metallic reinforcement in FiberFlex30D provided a better connection between filaments, thus changing the area of destruction (Figure 6h).

**Figure 7:**
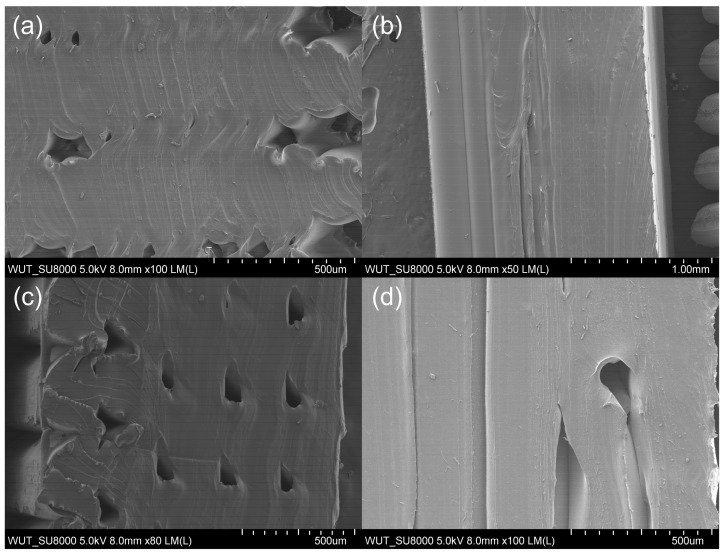
Cross-sectional microstructure of a FiberFlex30D sample before rupture: (**a**) across the filament without reinforcement, (**b**) along the filament without reinforcement, (**c**) across the filament with reinforcement, (**d**) along the filament with reinforcement.

Cross-sections of samples before and after rupture were assessed using SEM to find out how filaments were related to each other spatially. We also checked for any structural discontinuities. FiberFlex30D samples before rupture were examined as sections across (Figure 7a,c) and along (Figure 7b,d) filaments. All samples studied had a porous structure with cross-sectional voids. In samples with metallic reinforcement, the stainless steel wire was visible.

**Figure 8:**
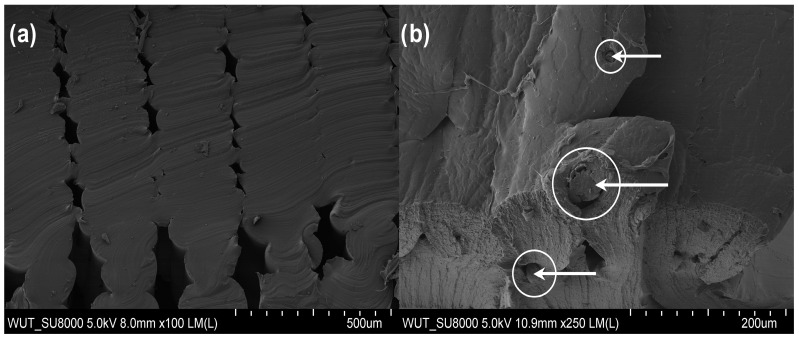
Cross-sectional microstructure of a FiberFlex30D sample: (**a**) without reinforcement, (**b**) with metallic reinforcement.

**Figure 9:**
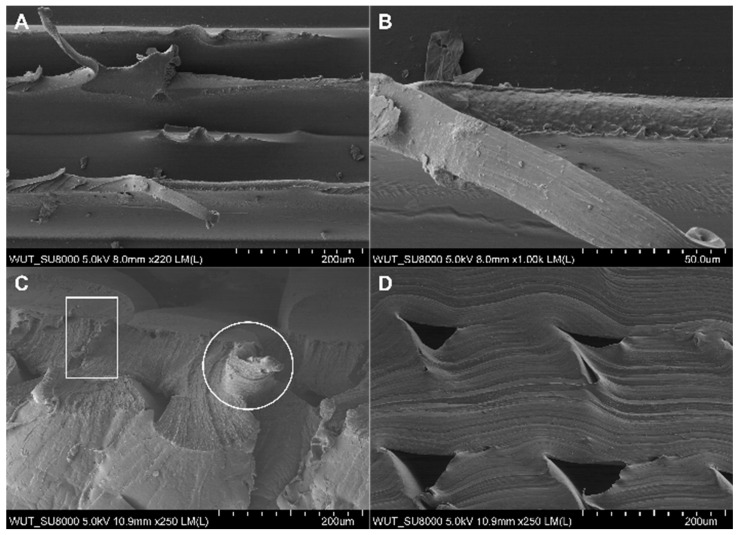
Microstructure of the rupture area of a FiberFlex30D sample: (**A**,**B**) without reinforcement, (**C**,**D**) with reinforcement.

In the case of samples after the tearing test, SEM images clearly show loose connection areas in the polymeric material. Moreover, the shape of individual elements can be seen in the cross-section (Figure 6a). Additionally, in the case of reinforced material (Figure 8b), a metallic wire is visible in the structure, marked with arrows in the figure. As a result of the unstable connection of the filaments and the applied force, the tear propagated at the inter-filament boundary (Figure 9A,B). In contrast, in the case of the FiberFlex30D material with metallic reinforcement, the SEM image (Figure 9C) shows that the rupture is both ductile (indicated by a square) and brittle (indicated by a circle). No reinforcement was found in the cross-section of the neck area. This indicates that the reinforcement broke early and was not involved in the process of neck elongation upon applying tensile stress. As in the case of the unreinforced material, voids are visible in the cross-section (Figure 9D).

**Figure 10:**
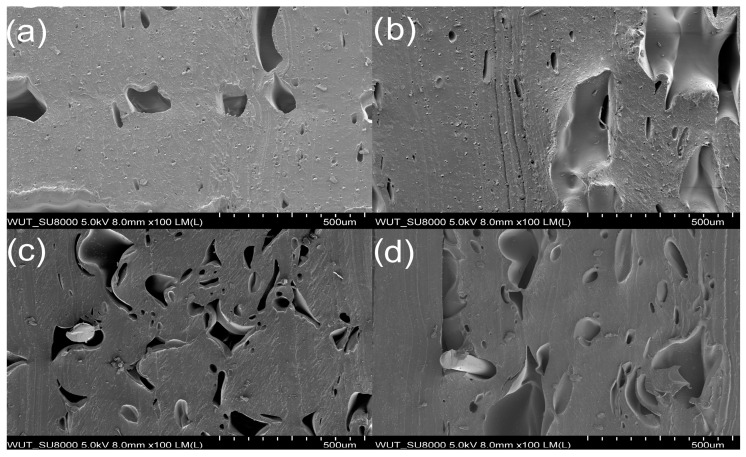
Microstructure of a sample of thermoplastic polyurethane (TPU95A) before rupture: (**a**) cross-section of a filament without reinforcement, (**b**) longitudinal section of a filament without metallic reinforcement, (**c**) cross-section of a filament with reinforcement, (**d**) longitudinal section of a filament with reinforcement.

In the case of TPU95A samples before rupture, filament cross-sections (Figure 10a,c) and longitudinal sections (Figure 10b,d) were examined. All images reveal a porous structure with cross-sectional voids, probably resulting from incomplete filament fusion in the process of 3D printing. SEM images also show particulate fillers in the TPU95A sample. The particles have an oval shape and dimensions up to 10 μm. In samples with metallic reinforcement, embedded stainless steel wire is visible.

**Figure 11:**
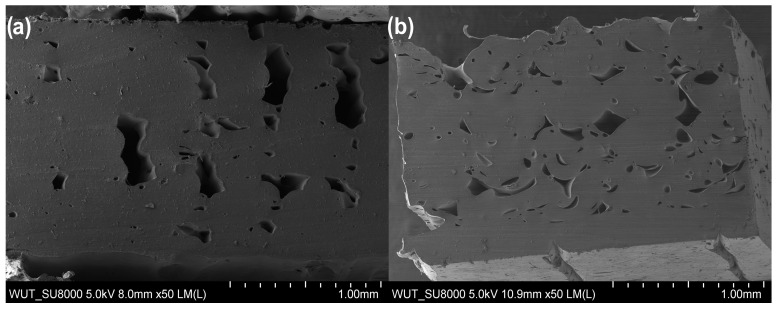
Post-rupture cross-sectional microstructure of a thermoplastic polyurethane (TPU95A) sample: (**a**) without reinforcement and (**b**) with reinforcement.

**Figure 12:**
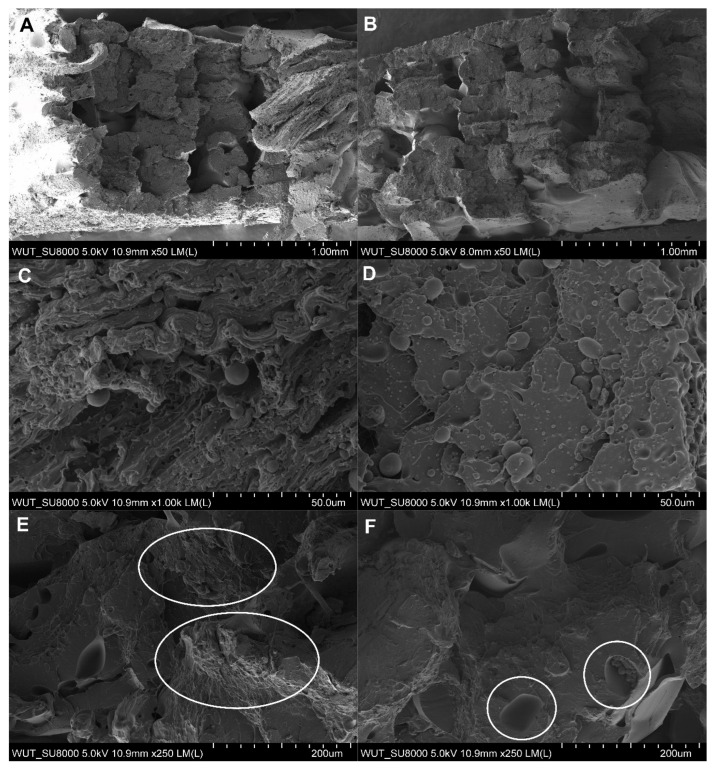
Microstructure of the rupture area of a thermoplastic polyurethane (TPU95A) sample: (**A**–**D**) without reinforcement and (**E**,**F**) with reinforcement.

Examination of post-tear TPU95A cross-sections showed voids (Figure 11a), which most likely occurred due to an unstable interfilament connection. Similarly, voids were noted in the material containing metallic reinforcement (Figure 11b). However, their size was smaller as compared to the unreinforced material. In addition, the fracture areas of the samples were also examined, which allowed for the assessment of the tensile mechanism. The microstructure of the rupture area of the TPU95A sample was found to be complex (Figure 12A,B). SEM images clearly showed elongated and permanently deformed outer filaments (Figure 12C), while the central part of the sample revealed brittle fracture (Figure 12D). Moreover, spherical elements with a diameter of up to 10 μm were found in the structure. A similar cracking pattern was observed for the TPU95A sample containing metallic reinforcement. SEM revealed areas of both ductile (Figure 12E) and brittle fracture (Figure 12F), as indicated in the images.

## 4. Discussion

3D printing as a method of fabricating innovative materials has many advantages, including the ease and speed of making complex elements as compared to traditional production techniques [28,29]. The aim of the work was to prepare cut-resistant coatings for protective gloves with metallic reinforcement using 3D printing technology. The texture design of 3D-printed materials is an important method of fine-tuning their mechanical properties. The proposed method aims to improve cut resistance and mechanical properties of gloves [30,31]. An important direction of research is the development of new composites that could replace the expensive and hard-to-produce ones that are now in use. Research on the application of stainless steel wire as reinforcement for polymeric materials adds to the design ideas and variety of 3D-printed products.

We demonstrated significant improvement in the cut resistance of the material incorporating stainless steel wire as compared to the same material without metallic reinforcement. The reinforcement increases energy absorption capacity, thus improving mechanical properties, including tensile strength [32,33,34,35]. The use of a 5 × 5 mm stainless steel mesh with a wire diameter of 0.04 mm could also have a positive impact on obtaining higher composite strength. Truong et al. [36] studied hybrid composites containing three different types of stainless steel mesh and showed that a fine mesh with stainless steel (0.3 mm) improved the mechanical properties of the studied materials. Moreover, improvement may be obtained by the arrangement of metallic reinforcement in both axial and transverse directions as a result of better interfacial adhesion [37].

It should be emphasized that the mechanical properties of the reinforced polymer composite depend not only on the properties of the reinforcement itself but also on those of the filament surrounding it as stresses are transferred between the two [38]. Similarly to other researchers, we believe that one of the most important parameters affecting the mechanical properties of composites is filament orientation. Longitudinally printed samples show better mechanical properties than those printed transversely [39]. The composites printed in our studies showed a well-ordered structure, and all elements were arranged parallel to the printing direction.

Another parameter influencing the mechanical properties of composites is the presence of voids in their structure. As can be seen from SEM images, the presence of metallic reinforcement reduces voids between adjoining filament layers. It seems likely that the voids formed along the metallic reinforcement are gradually filled, improving adhesion between individual filaments. Similar observations were made by Yu et al. [27], who printed polylactide (PLA) composites reinforced with basalt fiber. Moreover, Yu et al. distinguished two types of voids (interfiber and internal voids) affecting the strength of composites. It seems that despite the presence of voids in the entire structure of the composite materials, the high degree of orientation of filaments resulted in stronger materials than those obtained as a result of, for example, conventional pressing in a mold. It should be mentioned that the optimization of printing parameters, such as the adjustment of 3D printing speed or the amount of filament extruded through the nozzle, may reduce the formation of voids and improve the mechanical properties of the resulting composites.

The static tensile test study enabled a better understanding of the properties of composites containing metallic reinforcement. The improvement in interfacial bonding and energy absorption capacity was the primary reason for increased breaking strength and fracture strain. The types of microdamage during the tensile test mainly included filament elongation and ductile and brittle cracking. Most likely, filament fracture changes from ductile to brittle as the strain rate increases [40]. It was also important to incorporate metallic reinforcement inside the filaments. Placing reinforcements on the outer sides of the composite would reduce tensile and flexural strength while increasing tensile and flexural strains [15].

## 5. Conclusions

Several polymeric materials were successfully prepared using 3D printing technology. Their mechanical properties were assessed, and the most promising ones were selected. We demonstrated the feasibility of incorporating metallic reinforcement in the form of stainless steel wire in TPU95A and FiberFlex30D—two materials with different characteristics. It should be emphasized that the presence of metallic reinforcement improved the properties of the resulting hybrid materials. Cut resistance increased by 70%, and the force needed to tear the sample increased by over 20% compared to the pure polymer. Macroscopic examination clearly shows that mechanical strength depends on the type of polymeric material and adhesion between individual filaments, so improving the latter can enhance the former. Further research into optimizing printing parameters may indicate how to maximize the impact of filaments, minimize porosity, and increase mechanical properties [25,41]. Thus, we also showed the feasibility of using metal-reinforced polymeric materials as cut protection coatings for safety gloves. The study highlights the considerable impact of metal wire mesh reinforcement on the mechanical properties of hybrid polymer composites, providing valuable information for further research and practical applications.

## Figures and Tables

**Table 1:** Parameters of 3D printing of individual polymer materials.

Abbreviation	Print Speed [mm/s]	Fan Speed [%]	Bed Plate Temperature [°C]	Print Temperature [°C]
TPU 95A ^1^	25	20	70	235
FiberFlex 30D ^2^	45	25	60	240
PLA-CF 10% ^3^	60	100	60	220
PET-graphene ^4^	60	20	60	230
PLA ^2^	70	100	60	215
PETG ^2^	60	20	85	245
ABS ^2^	60	5	90	245

Producer: ^1^—Ultimaker, ^2^—Fiberlogy, ^3^—Spectrum, ^4^—Prografen.

**Table 2:** Calculation of cut resistance index.

Sequence	C_n_ Control Specimen	T_n_ Test Specimen	C_n+1_ Control Specimen	I Index
1	C_1_	T_1_	C_2_	i_1_
2	C_2_	T_2_	C_3_	i_2_
3	C_3_	T_3_	C_4_	i_3_
4	C_4_	T_4_	C_5_	i_4_
5	C_5_	T_5_	C_6_	i_5_

**Table 3:** Cut resistance requirements according to EN 388:2016+A1:2018.

Performance Level	Static Cut Resistance [a.u.]
1	1.2
2	2.5
3	5.0
4	10.0
5	20.0

**Table 4:** Performance levels assigned to the various cutting force thresholds according to EN ISO 13997:1999.

Performance Level	Level A	Level B	Level C	Level D	Level E	Level F
Cutting force [N]	2	5	10	15	22	30

**Table 5:** Polymer filaments with metallic reinforcement.

Abbreviation	Sample Photo	Macroscopic Photo of the Sample
TPU95A	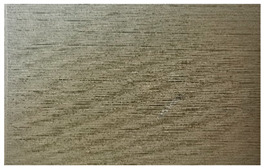	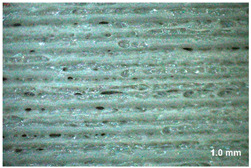
FiberFlex30D	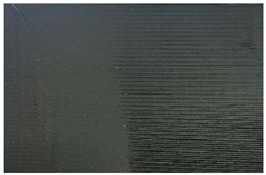	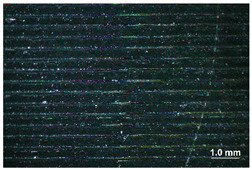

**Table 6:** Cut resistance results for the tested materials according to EN ISO 13997:1999.

Materials	Sample Thickness [mm]	Force [N]	Performance Level
FiberFlex30D	1.5	10.1	C
FiberFlex30D with reinforcement		14.8	C
TPU95A	1.5	12.8	C
TPU95A with reinforcement		21.9	D

## Data Availability

Data are contained within the article.

## Supplementary Materials

The following supporting information can be downloaded at: https://www.mdpi.com/article/10.3390/ma17010090/s1, Figure S1: Performance levels obtained by 0.5 mm thick samples in the static cut resistance test; Figure S2: Performance levels obtained by 1.5 mm thick samples in the static cut resistance test; Figure S3: Performance levels obtained by 0.5 mm thick samples in the dynamic cut resistance test; Figure S4: Performance levels obtained by 1.5 mm thick samples in the dynamic cut resistance test; Figure S5: Performance levels obtained by 0.5 mm thick samples in the abrasion resistance test; Figure S6: Performance levels obtained by 1.5 mm thick samples in the abrasion resistance test; Figure S7: Performance levels obtained by 0.5 mm thick samples in the puncture resistance test; Figure S8: Performance levels obtained by 1.5 mm thick samples in the puncture resistance test; Figure S9: Performance levels obtained by 0.5 mm thick samples in the tear resistance test; Figure S10: Performance levels obtained by 1.5 mm thick samples in the tear resistance test; Figure S11: Images of a TPU sample after tear testing; Figure S12: Images of a reinforced TPU sample after tear testing; Figure S13: Images of a FiberFlex sample after tear testing; Figure S14: Images of a reinforced FiberFlex sample after tear testing; Table S1: Information about individual polymer filaments based on safety data sheets from manufacturers; Table S2: Abrasion resistance criteria according to EN 388:2016 + A1:2019-01; Table S3: Puncture resistance criteria according to EN 388:2016 + A1:2019-01; Table S4: Tear resistance criteria according to EN 388:2016+A1:2019-01.
