# Supplementary material for: Functional 3D-Printed Polymeric Materials with Metallic Reinforcement for Use in Cut-Resistant Gloves
Source: Materials (Basel). 2023 Dec 23;17(1):90. doi: 10.3390/ma17010090 (PMC10779770; doi:10.3390/ma17010090)
Supplement: Supplementary file 1 [file materials-17-00090-s001.zip › materials-2771434-supplementary.pdf]

Supporting Information: Functional 3D printed polymeric materials with metallic reinforcement for use in cut-resistant gloves

E. Żyłka[1*], E. Irzmańska[1], J. Saramak[2], M. Jurczyk-Kowalska[3]

[1] Central Institute for Labour Protection – National Research Institute, Department of Personal Protective Equipment, Wierzbowa 48, 90-133 Lodz, Poland

[2] SMK3D Company, Opalowa 11/17, 93-487 Lodz, Poland

[3] Faculty of Material Science and Engineering, Warsaw University of Technology, Woloska 141, 02-507 Warsaw, Poland

***Corresponding authors:** emzyl@ciop.lodz.pl

Information about filaments

The bed plate temperatures used by SMK3D were generally consistent with those specified by the filament manufacturers. However, in the case of PLA-CF 10%, the bed plate temperature applied by the 3D printing company was higher by 15°C. In the case of print temperatures, the only discrepancy was observed for FiberFlex. The temperature used was 20°C higher than that specified by the manufacturer.

Table S1. Information about individual polymer filaments based on safety data sheets from manufacturers

Type of material	Abbreviation	Composition	Density [g/cm^3]	Print temperature [˚C]	Bed plate temperature [˚C]
Highly flexible rubber-like polymers	TPU	Thermoplastic polyurethane	1.22	-	-
	FiberFlex	FiberFlex 30D	1.07	200-220	50-70
Composites	PLA-CF 10%	Polylactic acid - carbon fibers 10%	1.30	190-220	0-45
	PET-graphene	Polyethylene terephthalate - graphene	1.29	220-250	55-60
Others	PLA	Polylactic acid	1.24	220-230	50-70
	PET-G	Polyethylene terephthalate glycol	1.27	220-250	90
	ABS	Acrylonitrile butadiene styrene	1.04	250-265	90-110

Static cut resistance

The static cut resistance test could not be performed for all 0.5 mm thick polymeric samples as it was impossible to mount PET-graphene, PET-G and ABS samples in the holder due to their high stiffness. In the case of the tested materials, the highest performance level of 5 was achieved by PLA, followed by level 3 obtained by the sample containing 10% carbon fibers (PLA-CF 10%).

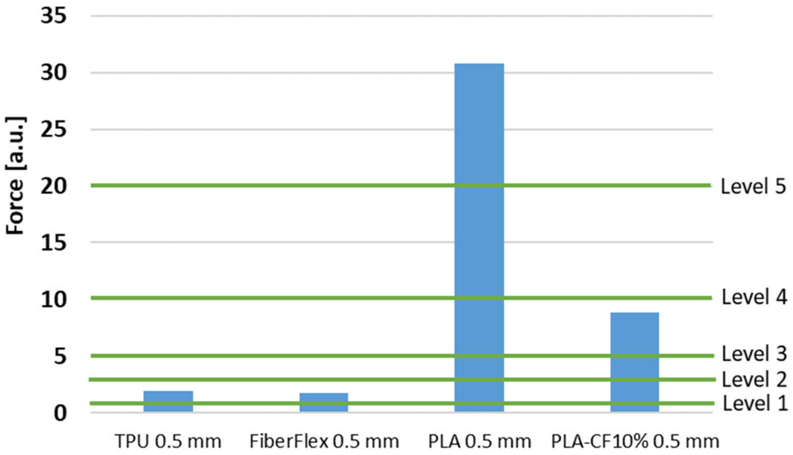

Figure S1. Performance levels obtained by 0.5 mm thick samples in the static cut resistance test.

For the same reason (high stiffness), the test could not be performed for most of the 1.5 mm polymeric samples – it was conducted only for TPU and FiberFlex. The TPU sample achieved performance level 3, while FiberFlex considerably exceeded performance level 2.

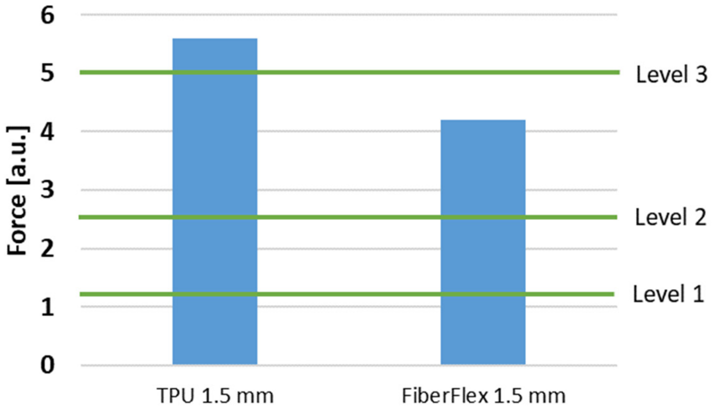

Figure S2. Performance levels obtained by 1.5 mm thick samples in the static cut resistance test.

Dynamic cut resistance

The dynamic cut resistance test was performed for all 0.5 mm thick polymeric samples. The lowest performance level of A was obtained by FiberFlex. The next performance level (B) was achieved by the TPU sample. All other tested polymeric materials met the criteria of the highest performance level (F). It should be emphasized that this level was achieved due to the high stiffness of the tested materials.

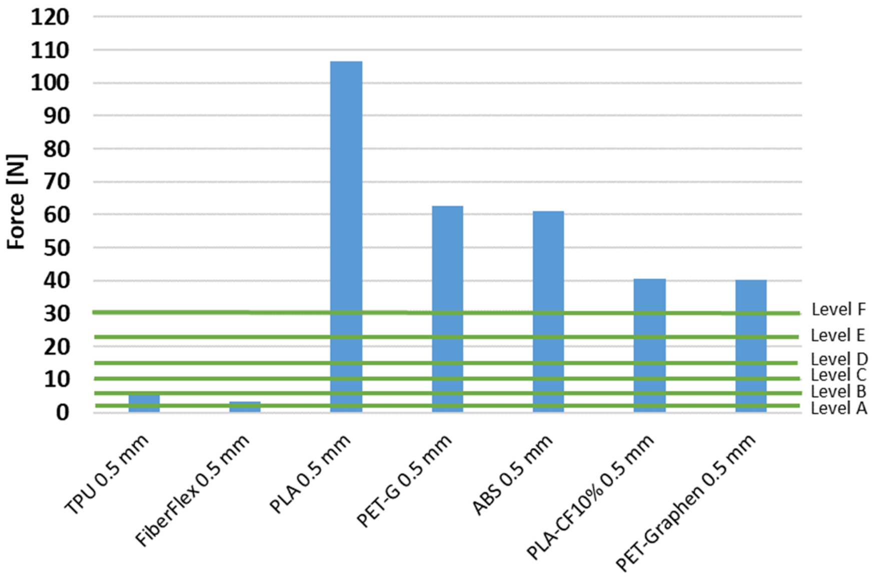

Figure S3. Performance levels obtained by 0.5 mm thick samples in the dynamic cut resistance test.

Due to the high stiffness of most of the studied materials, only TPU and FiberFlex 1.5 mm thick samples could be tested, as they retained sufficient flexibility. Both achieved performance level C, with the TPU sample considerably exceeding this level.

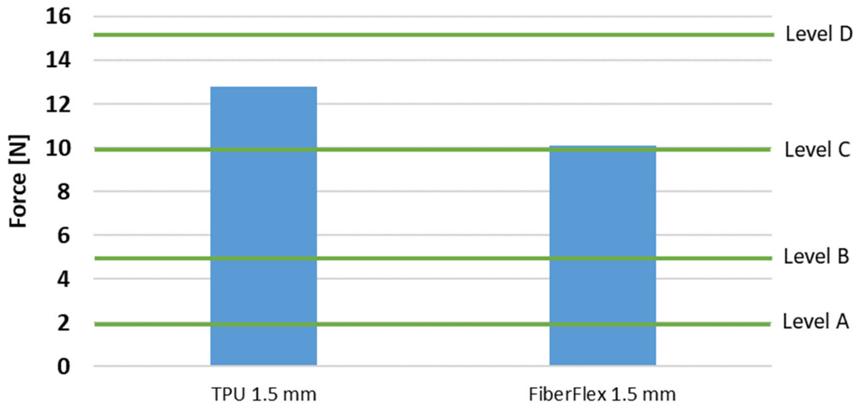

Figure S4. Performance levels obtained by 1.5 mm thick samples in the dynamic cut resistance test.

Abrasion resistance

Abrasion resistance was evaluated according to the standard PN-EN 388+A1:2019-01. The samples were placed in a specimen holder on the surface of the abradant. A weight was placed on each holder, exerting a pressure of (9 ± 0.2) kPa on the sample. The result of the abrasion resistance test was determined on the basis of changes on the surface of the abraded sample. If a breakthrough was found when examining a specimen at a given performance level, it was classified at the preceding level. Performance levels were assigned to the numbers of cycles according to Table S2.

Table S2. Abrasion resistance criteria according to EN 388:2016 + A1:2019-01.

Performance level	Abrasion resistance (number of cycles)
1	100
2	500

3	2000
4	8000

The test was performed for all polymeric materials. The lowest performance level of 1 was achieved by the sample containing 10% of carbon fibers (PLA-CF 10%). Level 2 was reached by the ABS sample, which was completely abraded. The same was true of the FiberFlex and PLA samples, which were classified at level 3. In the case of the TPU sample, which achieved performance level 3, clear breaks in the structure were observed. PET-G and PET-graphene were found to meet the criteria of the highest performance level (4). Their similar properties may result from the fact that both of these materials are thermoplastic polymers.

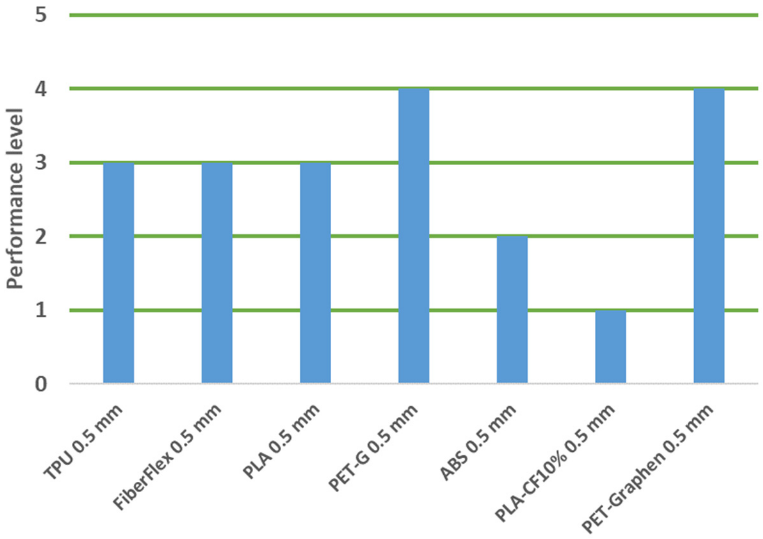

Figure S5. Performance levels obtained by 0.5 mm thick samples in the abrasion resistance test.

In the case of most 1.5 mm thick samples, the test turned out to be impossible to conduct due to the very high stiffness of the polymeric materials and the inability to mount them in the holder. Abrasion resistance was testable for two samples only: TPU and FiberFlex, both of which achieved performance level 4. None of the two tested samples was completely destroyed, only minor breaks in the structure of the polymeric material were observed.

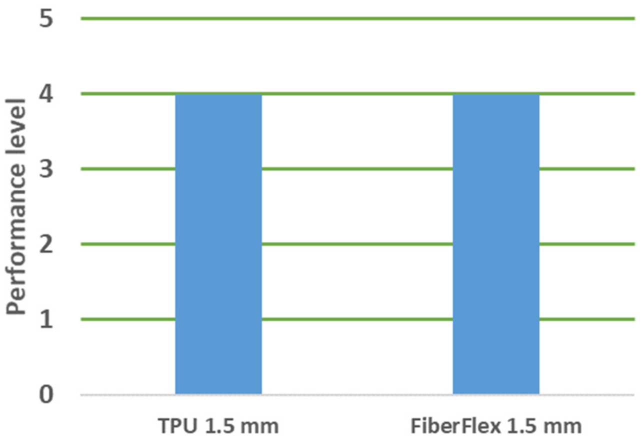

Figure S6. Performance levels obtained by 1.5 mm thick samples in the abrasion resistance test.

Puncture resistance

Puncture resistance was assessed in accordance with the standard EN 388:2016 + A1:2019-01. The testing machine was set to a compression rate of 100 ± 10 mm/min. Trials were carried out until the stylus completely pierced the samples. Performance levels were assigned to the different ranges of force used to puncture the samples according to Table S3.

Table S3. Puncture resistance criteria according to EN 388:2016 + A1:2019-01

**Performance level**	**Puncture resistance [N]**
1	20
2	60
3	100
4	150

The test was performed for all 0.5 mm thick polymeric materials. Two of the tested samples, TPU and FiberFlex, did not meet the requirements of the standard PN-EN 388+A1:2019-04. The remaining materials achieved performance level 1. The puncture resistance threshold for level 1 was considerably exceeded by PLA and PET-graphene samples.

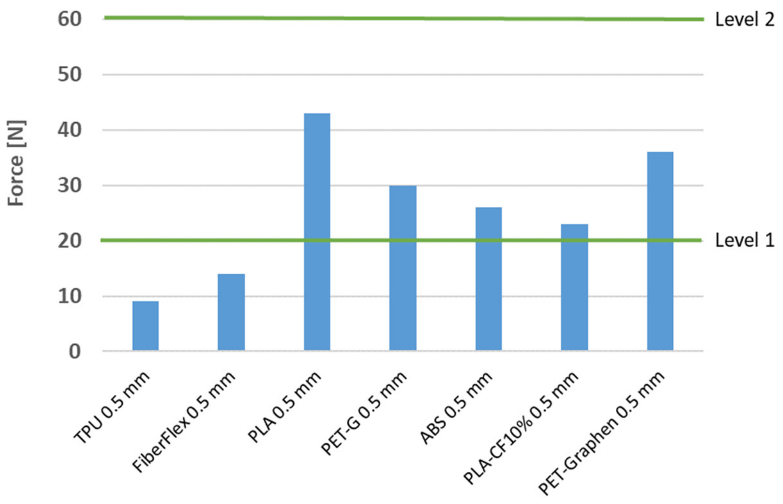

Figure S7. Performance levels obtained by 0.5 mm thick samples in the puncture resistance test.

The test was also performed for all 1.5 mm thick materials. One of the tested samples, i.e., FiberFlex, did not meet the requirements of the standard PN-EN 388+A1:2019-04. Performance level 1 was achieved by TPU, which actually came close to level 2. It should be emphasized, however, that most of the tested samples achieved the highest performance level of 4, while the ABS sample fell short of it only very slightly and was assigned to level 3.

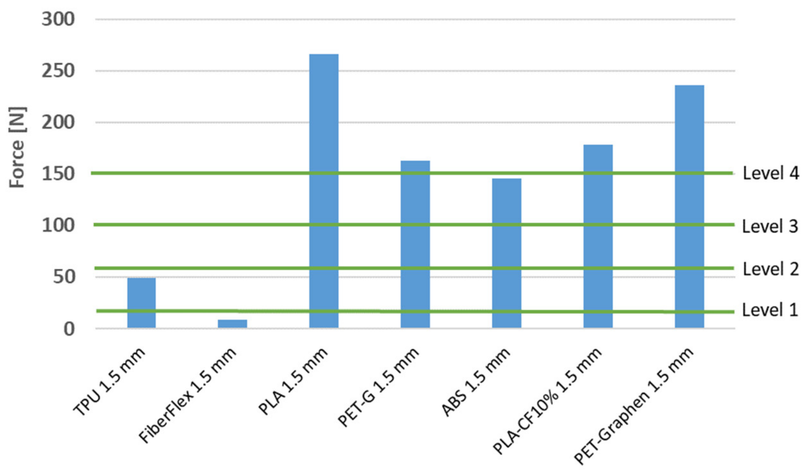

Figure S8. Performance levels obtained by 1.5 mm thick samples in the puncture resistance test.

Tear resistance

Tear resistance was evaluated in accordance with the standard EN 388:2016+A1:2019-01. The samples used in the test measured (100 ± 10) mm × (50 ± 5) mm and had incisions made in the longitudinal direction (25 ± 2.5) mm from their long edges. Tear resistance was expressed as the value of the tearing force [N]. Performance levels were assigned to tearing force values according to Table S4.

Table S4. Tear resistance criteria according to EN 388:2016+A1:2019-01.

Performance level	Tear resistance [N]
1	10
2	25
3	50
4	75

The test was carried out for all 0.5 mm thick polymeric materials except ABS, as the sample was too stiff to be properly mounted in the holder. Most materials fell short of the standard PN-EN 388+A1:2019-02, failing to achieve even the first performance level. The only exception was the TPU sample.

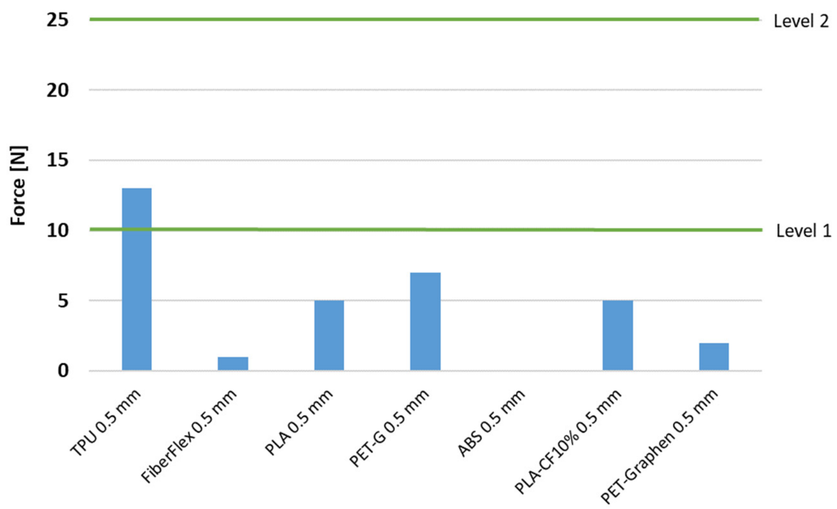

Figure S9. Performance levels obtained by 0.5 mm thick samples in the tear resistance test.

Due to the high stiffness of most of the tested materials, the test for 1.5 mm thick samples could be carried out only for TPU and FiberFlex. The remaining materials could not be properly clamped in the holder, and some cracked during mounting. The two successfully tested samples considerably exceeded performance level 1; they exhibited very similar tear resistance values.

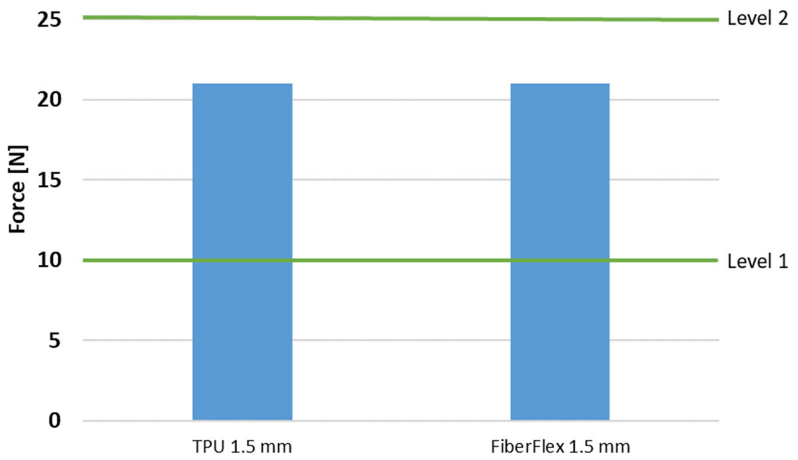

Figure S10. Performance levels obtained by 1.5 mm thick samples in the tear resistance test.

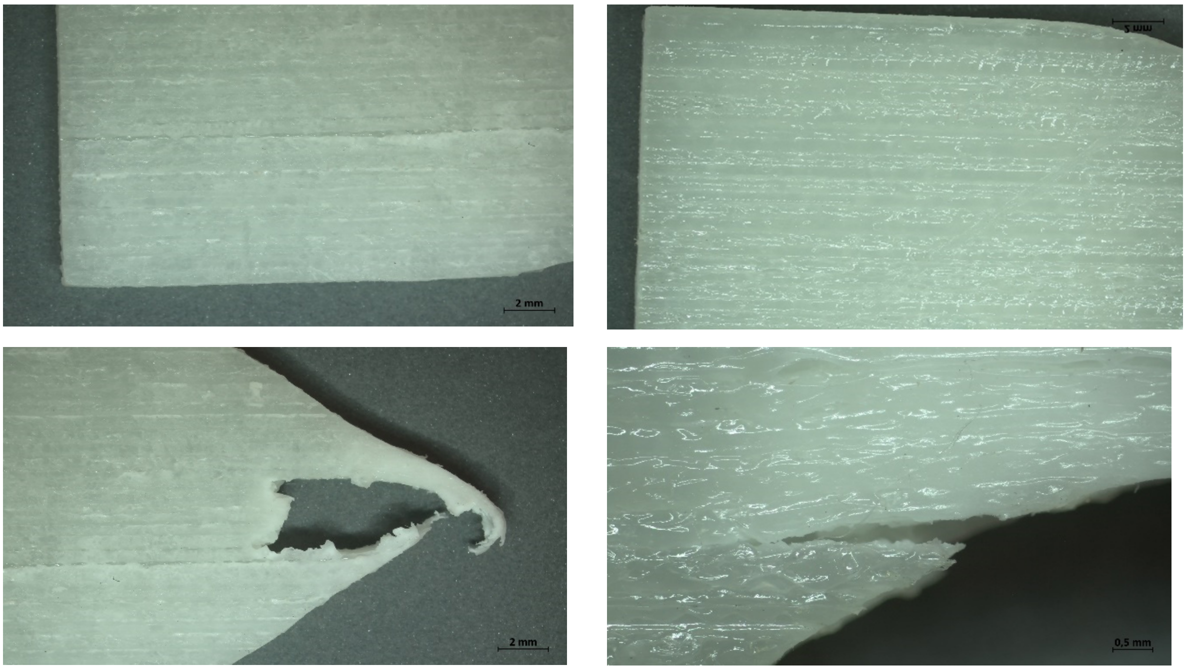

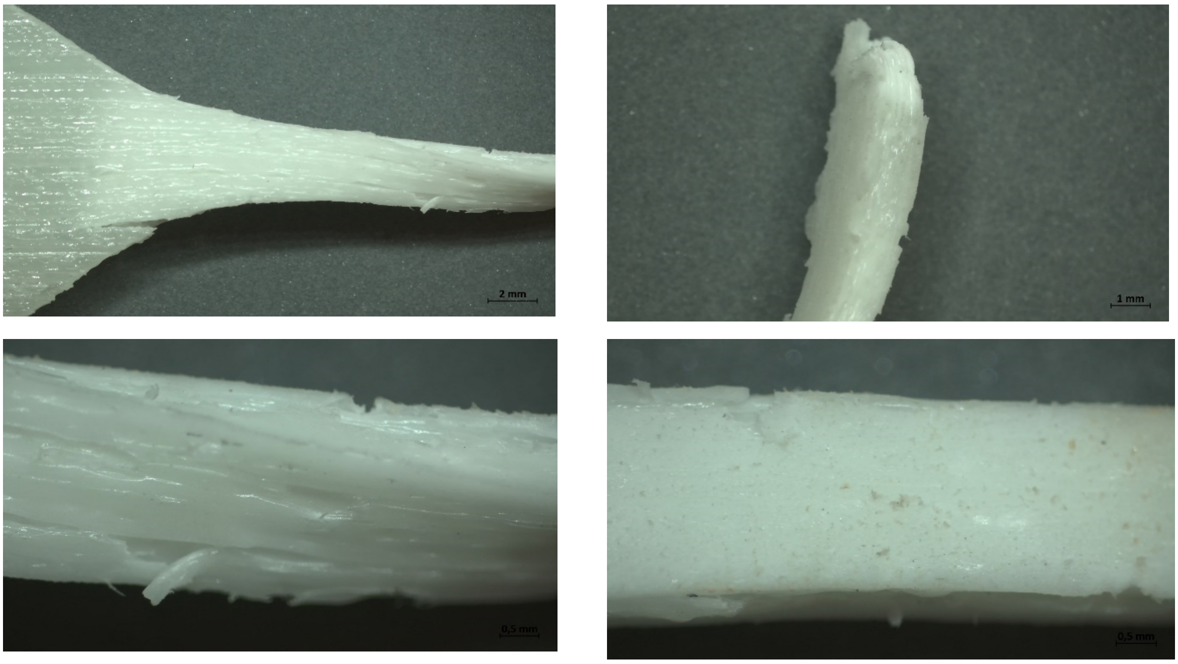

Figure S11. Images of a TPU sample after tear testing

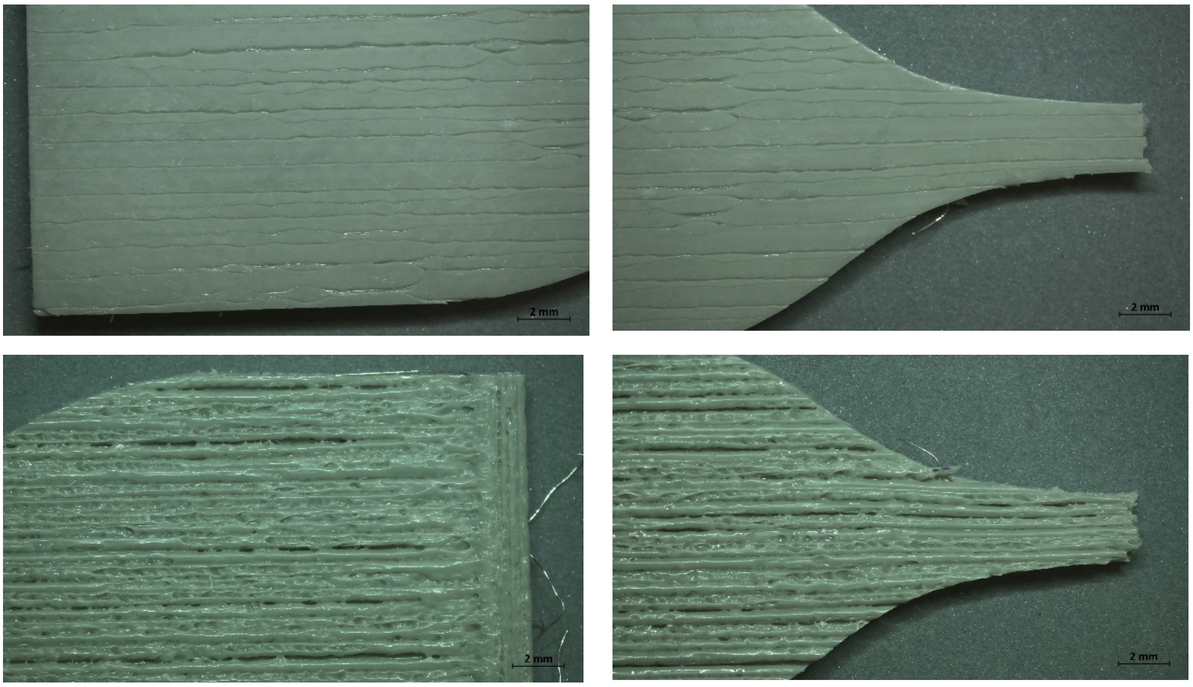

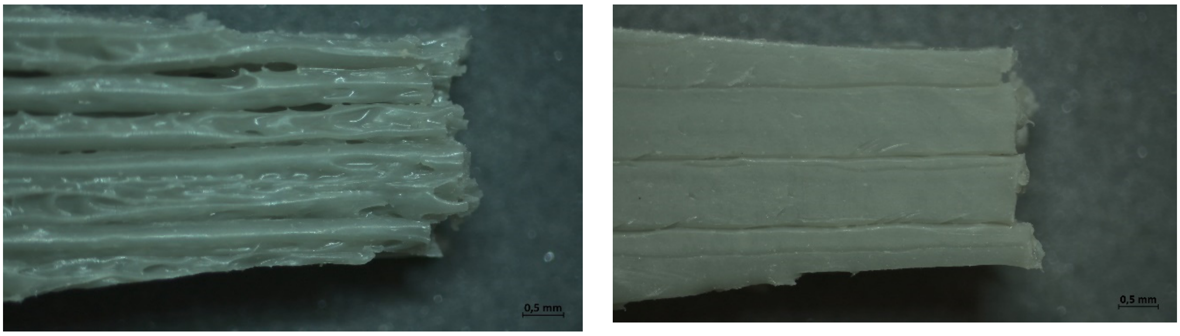

Figure S12. Images of a reinforced TPU sample after tear testing.

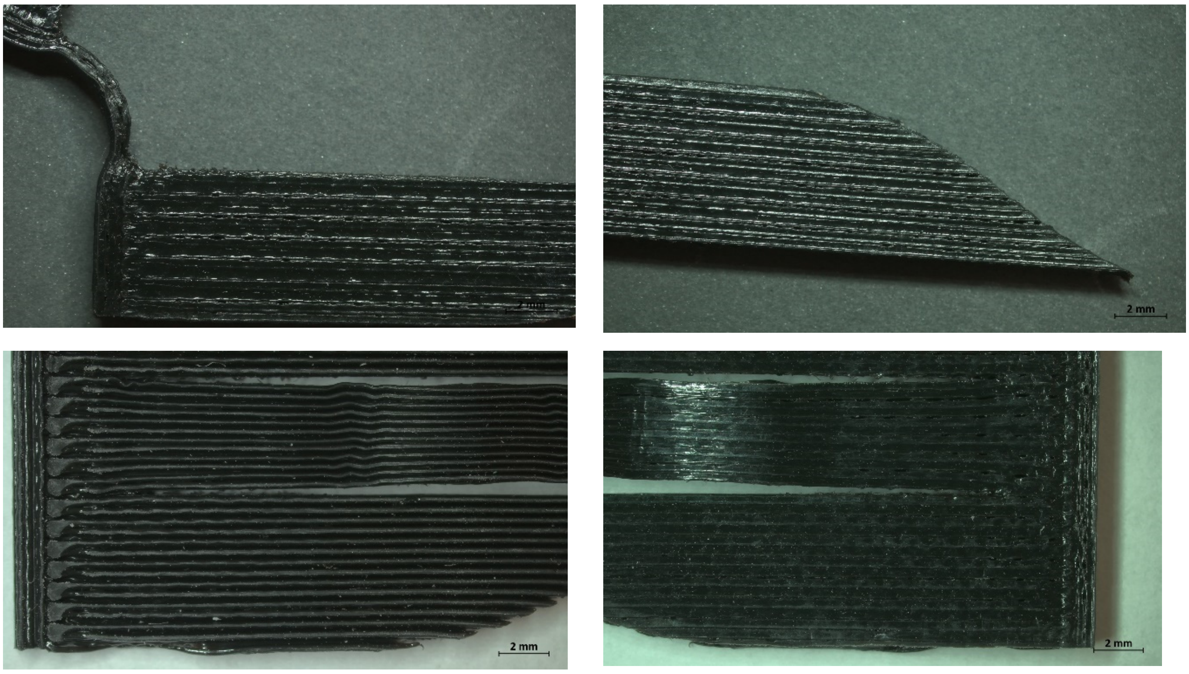

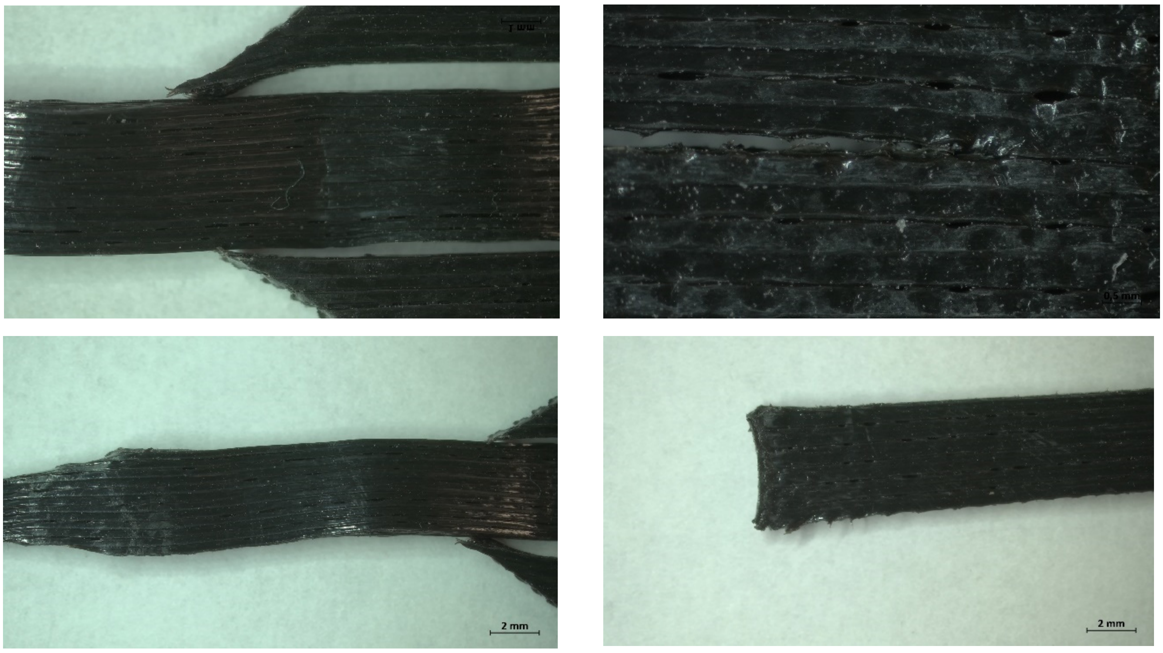

Figure S13. Images of a FiberFlex sample after tear testing.

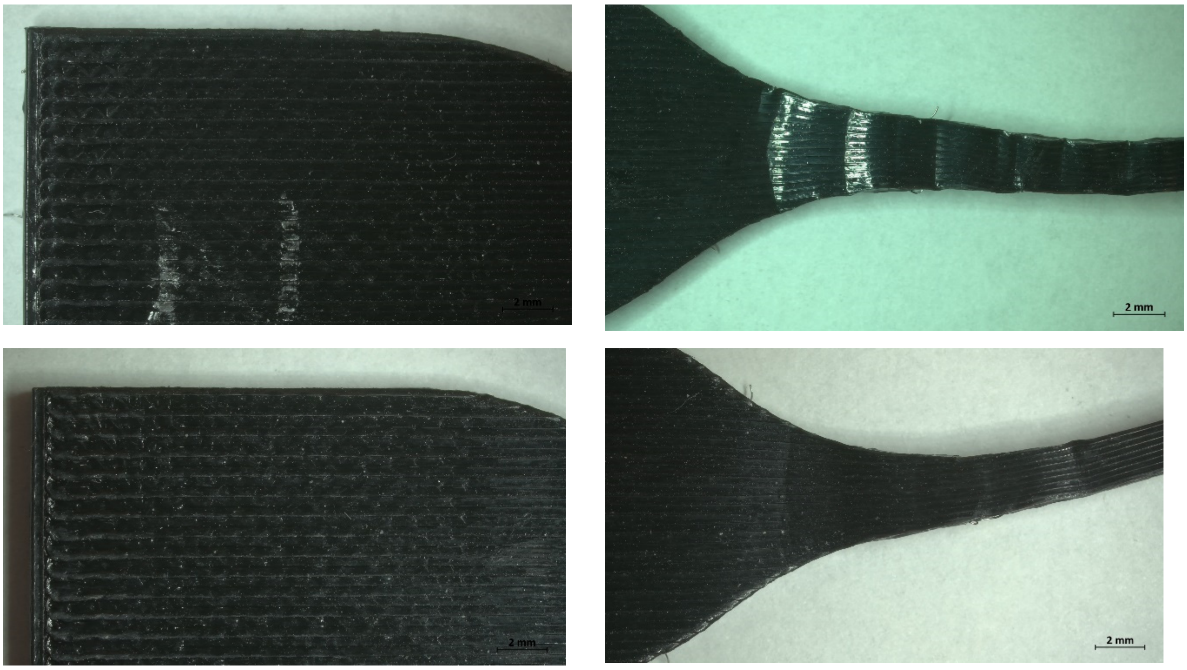

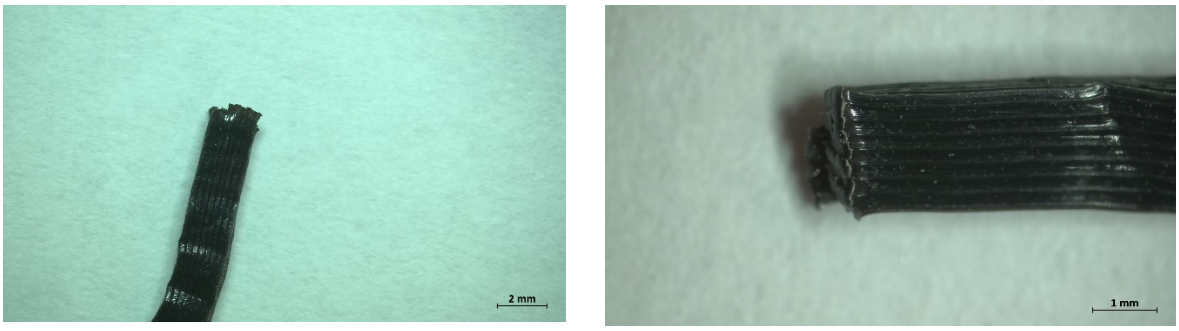

Figure S14. Images of a reinforced FiberFlex sample after tear testing.
